# Chronic Otomycosis Due to *Malassezia* Spp

**DOI:** 10.4103/0974-777X.62875

**Published:** 2010

**Authors:** R Latha, R Sasikala, N Muruganandam

**Affiliations:** *Department of Microbiology, Aarupadai Veedu Medical College & Hospital, Cuddalore Main Road, Kirumampakkam, Pondicherry-607 402, India*

**Keywords:** *Malassezia*, Otitis externa, Otomycosis

## Abstract

We report the case of a 31-year-old male presenting with complaints of mild pain in the right ear for three months and hypoacusis for 10 days. On otoscopic examination, a thin, papery, white material was extracted from his ear and sent for fungal identification. This material revealed presence of *Malassezia* spp – with characteristic “spaghetti and meat ball appearance”. The patient was treated with 2% acetic acid, hydrocortisone and Clotrimazole powder for one week and he resolved completely.

## INTRODUCTION

Otomycosis is an acute, subacute or chronic fungal infection of the pinna, external auditory meatus and the ear canal. Factors that predispose to otomycosis include absence of cerumen, humid climate, increased temperature, instrumentation of the ear and increased use of topical antibiotic/steroid preparations. *Aspergillus niger* and *Candida albicans* are the most common causative agents of otomycosis. Here we present an unusual case of chronic otomycosis caused by *Malassezia* species.

## HISTORY

A 31-year-old non-diabetic male presented with history of chronic otitis externa in the right ear for three months. He gave a history of using antibacterial eardrops on and off for last three months but with no effect. He complained of mild pain in the ear (on and off) for three months and hypoacusis for 10 days. He gave no history of tinnitus. On examination, the ear was dry with no discharge. Otoscopic examination revealed that the external meatus was plugged with abundant wax and was removed. After removal of the ear wax, a thin white papery material was extracted and was sent for fungal identification. Audiology examination showed he had a mild hearing loss of 25-30 db. The patient had no history of any other skin infection. He also gave history of having a pet dog at home.

**Figure 1:**
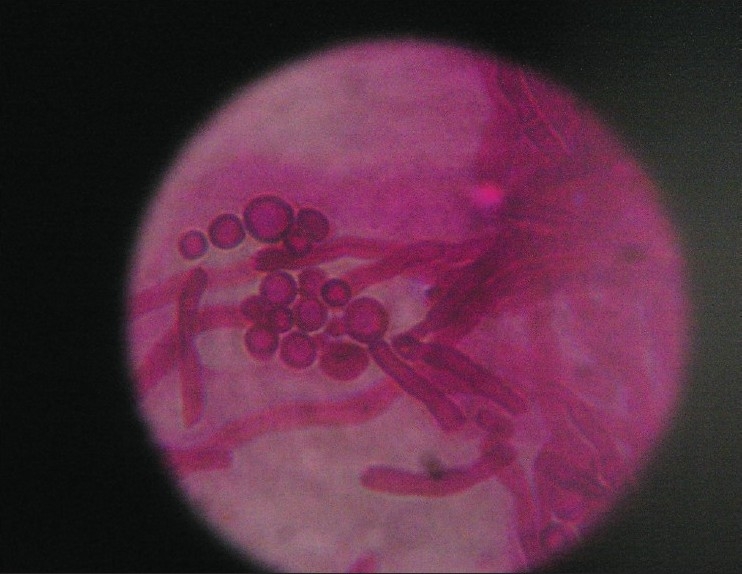
*Malassezia* species showing short hyphae and clusters of yeast cells occasional budding (Giemsa × 400)

The extracted white material was sent to the Microbiology department for fungal identification. The sample was examined under 10% KOH (Potassium Hydroxide) mount and inoculated on to SDA (Sabouraud's Dextrose Agar) for culture. 10% KOH mount revealed clusters of round yeast cells 2-7 μ in size with occasional budding. The hyphae were blunt, short, stout and curved- the characteristic “spaghetti and meat ball” appearance was observed, which was diagnostic of *Malassezia* spp. *Malassezia* spp. was identified based to their characteristic morphological features. Gram staining and Giemsa staining of the specimen also confirmed the same “spaghetti and meat ball” appearance. [Figure 1]. Several attempts were made to cultivate the organism on SDA overlaid with olive oil but all the attempts were futile.

## DISCUSSION

Although otomycosis has worldwide occurrence, its frequency is variable and depends on different climatic, occupational and socio-economic conditions.[1] Fungi are identified in about 10% of otomycosis cases.[2] The most common fungal etiological agents are *Aspergillus* spp. (80-90% of cases) and *Candida* spp.[3]

The genus *Malassezia* comprises a group of superficial fungi occurring as normal skin flora on the human body around the areas that are rich in sebaceous glands, but they are also known to cause infections or associated with certain skin diseases. Rarely, they become invasive and cause opportunistic infections under certain conditions. Their association with infection of external auditory canal (EAC) is uncommon however their role in the other human infections is gaining importance with the increase in the number of reports on *Malassezia* spp. infections in human.[4] Classically, prolonged treatment for bacterial otitis externa with topical antibiotics alters the flora of the ear canal and is also promycotic. Alterations in normal microflora in the ear and skin from prior or concurrent antibiotic therapy play a role as predisposing factors in allowing the overgrowth of *Malassezia* organisms.[3] Here, in this case report, history of prolonged use of antibacterial ear drops could be a possible explanation for otomycosis (*Malassezia*). However, reports also elucidate that fungus can occasionally be the primary pathogen, especially in the presence of excessive moisture or heat.[5] *Malassezia* spp. is commonly isolated from pet dogs and reports of otitis externa in dogs and cats are well known.[6,7] The source of infection for this patient is likely to have come from his pet dog. Dogs are the natural host for *Malassezia* spp., the potential for human exposure to the organism is therefore quite great and zoonotic transfer has been documented from dogs to immunocompromised patients by healthcare workers.[8] This supports our hypothesis that active *Malassezia* infection of canine skin or ear canals can become a risk factor for human carriage. Treatment of the patient was done in three steps; cleansing and debriding the ear of debris and lipid substrates, acidifying the canal with topical solution of 2% acetic acid and administration of antifungal agent clotrimazole powder for one week.[9] The patient resolved completely in two weeks.

## CONCLUSION

This case report indicates the importance and need for including *Malassezia* species in differential diagnosis of fungal otitis externa, especially with persons having close association with pet animals. This study also further reveals the indispensible importance of KOH examination in cases where the fungi involved is not easily cultivable. It is concluded that further detailed studies are necessary to confirm the transmission of *Malassezia* spp. from dogs to humans and role of *Malassezia* as an emerging pathogen.
